# Wearable Functional Near-Infrared Spectroscopy (fNIRS) Monitoring of Prefrontal Activation and Connectivity During Purpose-Driven Consumption

**DOI:** 10.3390/s26103097

**Published:** 2026-05-14

**Authors:** Daeun Kim, SuJin Bak, Sungkean Kim, Jaeyoung Shin

**Affiliations:** 1 Department of Applied Artificial Intelligence, Hanyang University, Ansan 15588, Republic of Korea; daeunkim@hanyang.ac.kr (D.K.); kimsk@hanyang.ac.kr (S.K.); 2 Advanced Institute of Convergence Technology, Suwon 16229, Republic of Korea; soojin7897@snu.ac.kr; 3 Department of Human-Computer Interaction, Hanyang University, Ansan 15588, Republic of Korea; 4 Department of AI Data Engineering, Korea National University of Transportation, Uiwang 16106, Republic of Korea

**Keywords:** fNIRS, social cognition, consumption, wearable neuroimaging

## Abstract

This study investigated the cortical activation patterns and functional connectivity underlying human decision-making by comparing two distinct purchasing orientations: other-oriented consumption (OOC) and self-oriented consumption (SOC), using functional near-infrared spectroscopy (fNIRS) as a wearable neuroimaging modality. The results revealed significant temporal concentration differences in ∆HbO under the OOC condition in Ch06 (p < 0.05). The 15 fNIRS channels were mapped to seven anatomically defined regions of interest (ROIs) to better capture regional activation patterns and functional network properties. While global network metrics showed no significant differences, seed-based connectivity analysis revealed that the OOC condition elicited significantly stronger functional connectivity between the medial prefrontal cortex (${ROI}_{4}$) and the left lower PFC (${ROI}_{6}$, p < 0.05, d = 0.45). In summary, while the overall network efficiency remained stable across conditions, our findings highlight a spatially specific enhancement in functional connectivity centered on the PFC, indicating an increased cognitive load from engaging in complex social cognitive processes. These findings advance the understanding of neural correlates underlying human decision-making and demonstrate the utility of wearable monitoring using fNIRS for capturing cognitive state differences in human-centered decision contexts.

## 1. Introduction

The purpose of a consumer purchasing a product varies depending on their needs and social environment. Generally, purchases are made for two main reasons: to acquire necessities for daily life and express identity or foster social relationships with others [1]. When purchasing behavior is categorized according to orientation, it can be broadly divided into other-oriented consumption (OOC) and self-oriented consumption (SOC) [2,3,4,5]. OOC refers to behaviors aimed at gifting others or enhancing social relationships [6]. These actions are influenced by external factors, such as others’ preferences, expectations, and aesthetic considerations. In this type of consumption, consumers make decisions based on the social context and the desire to strengthen their interpersonal connections. In contrast, SOC involves purchasing products to fulfill personal needs [7,8].

Unlike OOC, SOC is primarily centered on achieving personal satisfaction and fulfilling individual needs [9,10,11]. To investigate these two distinct consumption orientations, numerous researchers have predominantly relied on self-report methods to analyze consumers’ purchasing behaviors and decision-making processes based on their motivations [12]. Surveys are widely used self-reporting methods that employ structured questionnaires with quantitative scales to evaluate the various factors involved in consumers’ decision-making processes when purchasing products [13]. However, these methods rely on subjective responses, which introduce limitations such as social desirability bias and memory errors. Self-report is limited in its ability to determine whether the observed behavioral differences arise from preference inference for others, social appropriateness evaluation, or other neurocognitive processes. Therefore, neurophysiological measures offer an objective means to examine whether a shift in purchase goal is accompanied by distinct patterns of prefrontal activation and functional connectivity. This approach allows us to investigate whether the difference between OOC and SOC reflects a genuinely distinct neurocognitive process. From a consumer neuroscience perspective, the distinction between OOC and SOC may reflect a fundamental conflict between value-based evaluation and cognitive control within the prefrontal cortex (PFC). The ventromedial prefrontal cortex (vmPFC) has been shown to intensify its activity during preference judgments relative to perceptual discrimination tasks, suggesting its critical role in encoding subjective value [14]. The orbitofrontal cortex (OFC), a subregion of the PFC, is associated with the anticipation of rewards and has been consistently implicated in product selection and consumer choice [15]. In contrast, the dorsolateral prefrontal cortex (dlPFC) is recruited for cognitive control and the suppression of impulsive responses during decision-making [16]. Critically, when decisions involve a conflict between self-interest and normative considerations, such as when fairness and personal gain are in opposition, functional connectivity between the posterior vmPFC and the right dlPFC intensifies, enabling normatively aligned decisions even at a personal cost [17]. This suggests that OOC engages a fundamentally different neural architecture than SOC. The distinction is not merely a difference in behavioral preference, but reflects a conflict between self-oriented value signals and socially guided regulatory processes within the PFC.

Generally, OOC may involve complex neural network connectivity and higher cognitive functions as it requires predicting others’ preferences and considering social interactions through intricate social cognition and emotional evaluation [18,19,20]. By contrast, self-oriented consumption may rely more heavily on familiarity and processing fluency, facilitating seamless evaluation and potentially faster choice through established heuristics such as brand recognition, price, and perceived utility [21,22,23,24,25]. These two consumer decision-making processes are therefore expected to be associated with distinct patterns of brain activation and functional connectivity, which self-report and behavioral measures alone are insufficient to capture. Neuroimaging studies have shown that the mPFC plays a key role in representing self-importance and distinguishing self- from other-referential processing [26], and is specifically engaged when monitoring the discrepancy between self- and other-perspectives during social cognition tasks [27]. Moreover, decisions that consider others’ needs and desires consistently recruit the dorsal mPFC and temporoparietal junction [28], suggesting that OOC constitutes a neurally distinct form of socially contextualized decision-making. Through neuroscientific analysis, we aimed to examine the neural factors underlying these differences. This neuroscientific approach not only captures brain activation and functional connectivity but also provides an objective complement to self-report methods.

Functional near-infrared spectroscopy (fNIRS) is a valuable technology for classifying activation patterns in the cerebral cortex [29]. This allows researchers to identify various cerebral regions in a noninvasive manner. Electroencephalography (EEG) and functional magnetic resonance imaging (fMRI) have been widely used in neuroscience research [30,31,32,33]. Despite its excellent temporal resolution, conventional electrode recordings still require skin preparation and conductive gel, thereby increasing setup time and participant burden during experiments. While recent advancements in portable dry electrode technologies have begun to overcome their susceptibility to electrical noise and motion artifacts, these systems often still require a complex setup process that increases both setup time and participant burden in the experiment. fMRI provides superior spatial resolution and enables observation of brain activation patterns across all brain regions. However, its high costs and spatial constraints hinder its use in neuroscientific research. In contrast, fNIRS offers both portability and practicality, making it ideal for real-life research [29]. fNIRS does not require the use of a conductive gel and is minimally constrained by spatial limitations. Therefore, it can be considered a suitable neuroimaging technology for conducting research closely related to real-life applications [34,35]. Accordingly, fNIRS was selected as the primary tool for the present study to investigate consumer decision-making in a naturalistic setting. The suitability of fNIRS for consumer research has been demonstrated in various neuroscience studies [36,37,38]. As a portable bioelectronic sensing device, fNIRS can be regarded as a soft bioelectronic sensing approach for human-centered applications. In the present study, we apply this wearable sensing technology to a consumer decision-making task in a naturalistic context, thereby extending its relevance to human-centered behavioral research beyond conventional laboratory settings.

As previously mentioned, this study focused on identifying differences in brain activation patterns and functional connectivity between two types of purchasing behavior with distinct orientations in the PFC. The PFC was selected because it is consistently associated with subjective valuation and cognitive control within the context of socially contextualized decisions [39,40]. The present study extends prior work conducted with the same dataset by incorporating functional connectivity and network-level analyses to provide a more comprehensive characterization of the neural mechanisms [41]. While regional activation analyses can identify which brain areas are engaged during a given task, they do not capture the dynamic coordination between regions that underlies complex decision-making. Given that OOC involves the integration of reward-based value signals in the vmPFC and OFC, and normative regulatory processes in the dlPFC, examining functional connectivity beyond activation alone offers a more comprehensive understanding of how these distinct processes are coordinated in real time.

Furthermore, we discuss the underlying mechanisms that cause these differences from cognitive and emotional perspectives. Specifically, we investigated brain activation patterns and functional connectivity in the PFC induced by purchasing decision-making tasks using fNIRS technology. The remainder of this paper is organized as follows. The research methods are detailed in Section 2, and the results are presented in Section 3. The findings are discussed in Section 4, and the conclusions are provided in Section 5.

## 2. Methods

### 2.1. Participants

The sample size was determined via an a priori power analysis conducted with G*Power 3.1 [42]. Assuming an effect size of $d_{z}=0.5$, α = 0.05, and power = 0.90 [43], the minimum required sample size was estimated at *N* = 44. To account for potential fNIRS data loss based on predefined signal-quality criteria, a total of 50 healthy volunteers were initially recruited for this study. Among them, 6 participants were excluded from the final analysis due to low signal-to-noise ratio and an insufficient number of valid channels for reliable preprocessing and analysis. Therefore, the final analysis involved 44 healthy volunteers (25 men and 19 women with an average age of 22.8 years ranging from 19 to 37 years). The detailed demographic information is presented in Table 1. All individuals provided informed consent before participation, and none reported a history of neurological or psychiatric disorders. This study was approved by our Institutional Review Board of Wonkwang University (No. WKIRB-202405-HR-023) and the Declaration of Helsinki [44].

### 2.2. fNIRS Data Acquisition

**Figure 1:**
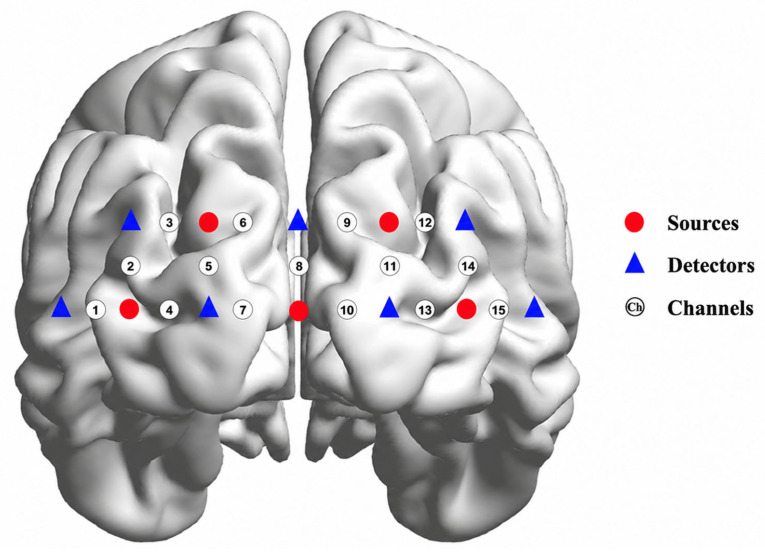
fNIRS 15 channels on the prefrontal cortex.

The experiment was conducted using an fNIRS device (NIRSIT LITE, OBELAB Inc., Seoul, Republic of Korea) to record changes in oxygenated (∆HbO) and reduced (∆HbR) hemoglobin concentrations in the PFC region with a sample rate of 8.138 Hz [45]. This sampling rate reflects the fixed acquisition setting of the hardware. The fNIRS device consisted of 15 channels positioned over the participants’ foreheads to measure both ∆HbO and ∆HbR in the PFC. The inter-optode distance between each adjacent source and detector pair was maintained at 3 cm. Figure 1 shows the placement of the fNIRS optodes on the prefrontal cortex, with an arrangement of 15 channels. The MNI coordinates and corresponding anatomical locations for each fNIRS channel are summarized in Appendix A.

### 2.3. Experimental Paradigm

**Figure 2:**
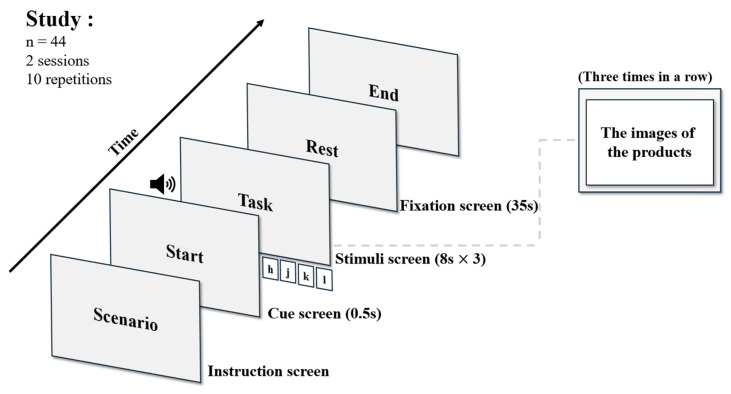
Experimental Paradigm. The figure illustrates the task structure that was used for both OOC and SOC conditions. In each trial, three stimulus screens were presented consecutively (8 s each), and participants selected one of four products on each screen using the keyboard (h, j, k, and l). The sound icon represents the auditory cue used to signal the beginning of the task period. The dashed line indicates the detailed content of the stimuli screen presented during the task.

To reduce motivational heterogeneity across conditions, stimuli were restricted to a single product category. Because beneficiary-driven decisions can interact with product type (e.g., hedonic vs. utilitarian) and thereby alter choice patterns, functional food products were selected as stimuli [46,47]. This category supports a goal-directed, utilitarian framing, minimizes the risk of self-related purchasing being interpreted as self-gifting, and enables a cleaner comparison between the two consumption orientations [48]. The experimental paradigm is illustrated in Figure 2. All tasks were programmed using the E-Prime 3.0 software (Psychology Software Tools Inc., Pittsburgh, PA, USA) and displayed on a 27-inch monitor. Task events and fNIRS signals were synchronized using event markers. Participants were asked to choose a product that they were willing to purchase under two conditions. OOC was operationalized as selecting a red ginseng gift set for a person who has provided emotional support during difficult times, thereby incorporating elements of gratitude, reciprocity, and social obligation. SOC was operationalized as selecting a red ginseng product as a reward for oneself following a period of physical fatigue, framed as self-care rather than self-gifting. The contextual differences between conditions are inherent to the respective constructs, as these motivational elements are theoretically embedded in the definitions of other- and self-oriented consumption [49]. The presentation order of the two conditions was randomized and counterbalanced across participants. For each condition, participants completed 10 trials. A single trial consisted of an introduction period (0.5 s), a task period (24 s), and a rest period (35 s). During the task period, three consecutive stimulus screens were presented for 8 s each. On each stimulus screen, four product options were displayed, and participants selected one option using the corresponding keys (h, j, k, l). Thus, each condition yielded 30 selections (10 trials × 3 stimulus screens). The products belonged to the same category of health-functional foods but varied in two dimensions: packaging and price. The packed products were priced higher, whereas the non-packed options were priced lower. During the rest period, a fixation cross was presented visually. The presentation order and on-screen positions of the product options were randomized across trials. Following the experiment, participants completed a self-report questionnaire assessing purchase intention, product satisfaction, and packaging preference using a 5-point Likert scale. Refer to Bak et al. [41] for a comprehensive description of the experimental paradigm and the results of this questionnaire.

### 2.4. Preprocessing

fNIRS data preprocessing was conducted using MATLAB R2023b (MathWorks, Natick, MA, USA). ∆HbO was used exclusively for analysis [50,51], as it demonstrates greater sensitivity and signal-to-noise ratio (SNR) compared to ∆HbR and is more widely adopted as the primary indicator of cortical activation in fNIRS research [52,53,54,55]. Optical intensity changes evoked by task execution were converted to ∆HbO using the modified Beer-Lambert law (MBLL). The converted fNIRS data were then resampled to 2 Hz to reduce computational cost without significant information loss. The fNIRS data were band-pass filtered using a zero-phase filter based on a third-order Butterworth filter with a passband of 0.005–0.05 Hz to remove the physiological noise and DC offset [56]. The filtered fNIRS data were segmented into epochs from −1 to 61 s relative to task onset (i.e., 0 s) and baseline-corrected by subtracting the average value within the reference interval of −1 to 0 s. For each condition, functional connectivity was analyzed using continuous segments of approximately 10 min in duration. All preprocessing steps were executed using the BBCI (The Berlin Brain–Computer Interface) toolbox to ensure efficient handling of fNIRS data for further analysis [57].

### 2.5. Functional Connectivity Measures

The synchrony of fNIRS signals was assessed using the phase-locking value (PLV), which is given by (1), where N and $\Delta \phi \left(t\right)$ denote the number of data points and the instantaneous phase difference between two signals, respectively. The PLV quantifies the phase synchrony between signals by evaluating the consistency of the phase differences across trials, employing the Hilbert Transform. The PLV, ranging from 0 to 1, represents the degree of synchrony between functional regions of the cerebral cortex within a specific frequency range.

(1) $$PLV=\left|\frac{1}{N}\sum_{t=1}^{N}e^{j\Delta \phi \left(t\right)}\right|$$

PLV was computed from the filtered fNIRS signals using the Hilbert transform to obtain instantaneous phases. For each participant, PLV was estimated over the entire continuous segment of each condition, yielding a connectivity matrix for all channel pairs. This duration provides sufficient data for stable phase-synchrony estimation within the target frequency band [58]. For ROI-level analysis, connectivity was defined as the mean PLV across all channel pairs linking two predefined regions of interest (ROIs). Network metrics were subsequently computed from the PLV connectivity matrix.

### 2.6. Regions of Interest (ROIs)

Based on these anatomical mappings [59], the channels were grouped into seven functional ROIs to capture regional activation patterns: ${ROI}_{1}$ (Lateral Right; Ch01 and 02), ${ROI}_{2}$ (Upper Right; Ch03, 05, and 06), ${ROI}_{3}$ (Lower Right; Ch04 and 07), ${ROI}_{4}$ (Center; Ch08), ${ROI}_{5}$ (Upper Left; Ch09, 11, and 12), ${ROI}_{6}$ (Lower Left; Ch10 and 13), and ${ROI}_{7}$ (Lateral Left; Ch14 and 15). The seven ROIs were used to construct an ROI-level connectivity matrix. Because ${ROI}_{4}$ corresponded to a central prefrontal region in the vicinity of the mPFC [60], seed-based connectivity analysis was conducted using ${ROI}_{4}$ as the seed and the remaining six ROIs as targets. Table 2 summarizes the seven ROIs and the seed-based connectivity analysis. This parcellation approach is consistent with searchlight-based probabilistic mapping methods used in prior fNIRS studies employing the same device [61].

### 2.7. Network Metrics

Network analyses were performed on the ROI-level connectivity matrix derived from PLV analyses. Weighted global efficiency was calculated to summarize overall network integration, and nodal strength was computed to quantify the total connectivity of each ROI. In addition, seed-based connectivity analysis was conducted using ${ROI}_{4}$ as the seed regions for the remaining ROIs. Local efficiency and clustering coefficient were also examined as supplementary network measures. The procedures mentioned above were implemented using the Brain Connectivity Toolbox to enhance computational efficiency and ensure the reproducibility of the results [62].

### 2.8. Behavioral Measures

In addition to fNIRS data, behavioral data were collected to examine the participants’ decision-making processes. The behavioral data included the duration of product selection and the frequency of selecting lower-priced products for each product under the two conditions. The frequency of selecting lower-priced products was calculated across trials to examine efficiency tendencies in each condition. A paired *t*-test was conducted to account for within-subject variability between OOC and SOC conditions. These analyses aimed to differentiate the participants’ product selection patterns based on conditions, highlighting the distinct decision-making strategies employed in each context.

## 3. Results

### 3.1. Brain Activations

**Figure 3:**
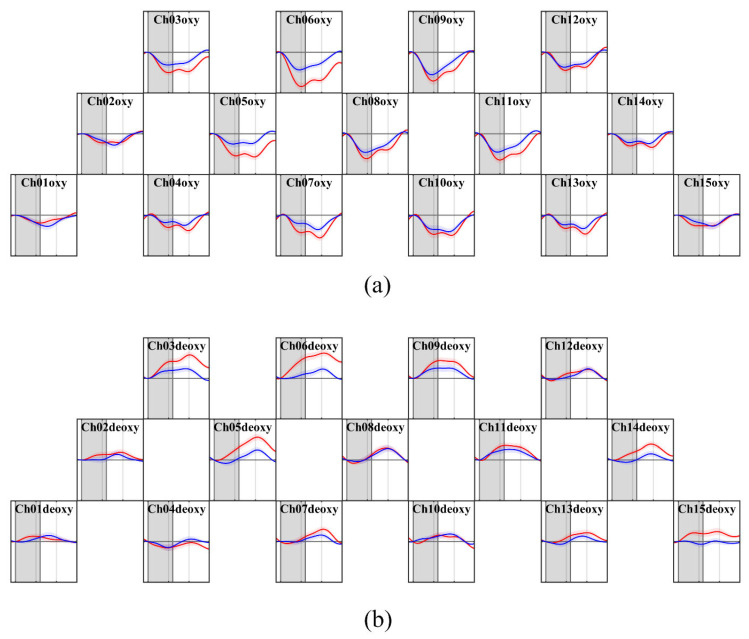
Grand averages of (**a**) ∆HbO (**b**) ∆HbR across all participants for each condition (red: OOC, blue: SOC). Shaded areas around the solid lines represent the standard error of the mean. Grey shaded regions indicate task blocks (24 s each); white regions indicate rest periods. ∆HbR is presented for reference only and was not included in the primary analysis (*x*-axis: s; *y*-axis: μM).

The mean hemodynamic response differences in brain activation for each participant were analyzed according to the gift-giving conditions. Figure 3 illustrates the grand averages of temporal concentration changes of ∆HbO/R across for all participants in both conditions. The t-test results for temporal concentration changes in ∆HbO revealed a significantly greater response under OOC condition than SOC condition in Ch06 (t(43) = 3.14, p = 0.045, after FDR correction, d = 0.47, Benjamini–Hochberg method, q = 0.05).

### 3.2. Functional Connectivity Analysis

**Figure 4:**
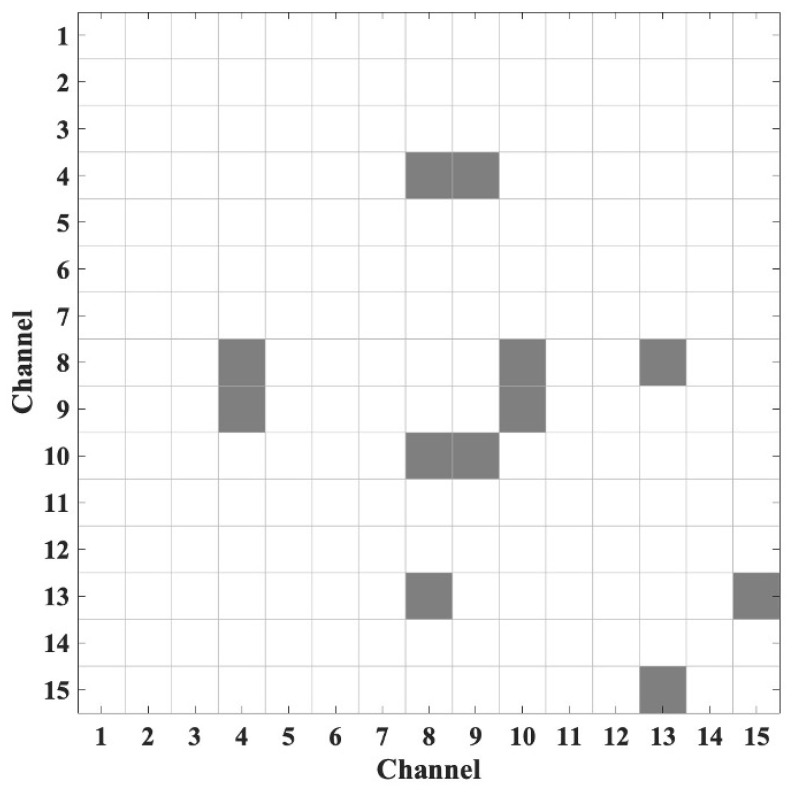
Grand average of heatmap showing statistical differences in phase synchronization between the two conditions, where significant differences are represented in gray and non-significant differences are shown in white.

**Figure 5:**
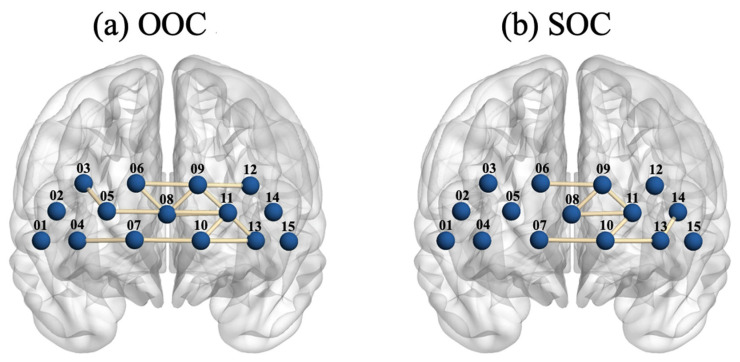
Grand average of binary connectivity maps for both conditions. (**a**) OOC (**b**) SOC. In each map, nodes represent the 15 fNIRS channels placed over the prefrontal cortex with edges indicating functional connectivity (Threshold = 0.7).

Figure 4 illustrates the functional connectivity matrix derived from PLV analyses for the OOC and SOC conditions. The heatmap highlights statistically significant differences in phase synchronization between the two conditions, where gray cells represent channel pairs with significant functional connectivity (p < 0.05) and white cells indicate no significant difference. The matrix indicates that the OOC condition exhibits stronger phase coupling than the SOC condition, reflecting more robust neural synchrony. Figure 5 complements this by presenting binary connectivity maps for each condition separately, in which only channel pairs with absolute PLVs exceeding 0.7 are displayed. This threshold was applied solely for visualization purposes to highlight strong connections. Figure 4 tests between-condition statistical differences, whereas Figure 5 visualizes within-condition connectivity strength.

### 3.3. Network Analysis

At the global network analysis, paired *t*-tests revealed no significant differences in global efficiency between the OOC and SOC conditions (t(43) = 1.38, p = 0.174, d = 0.32). Likewise, nodal metrics showed no statistically significant differences across all ROIs after FDR correction (Benjamini–Hochberg method, q = 0.05). In the seed-based connectivity analysis, the S5 connection showed significantly stronger connectivity under the OOC condition than under the SOC condition (p < 0.05, d = 0.45). A similar pattern was observed for the S3. However, this effect did not survive the multiple comparison adjustment (p = 0.12, d = 0.32).

### 3.4. Behavioral Data

First, a paired t-test was conducted to identify differences in the time spent selecting a product between the two conditions. Eighteen participants spent more time choosing a product in the OOC condition than in the SOC condition (p < 0.05) (Table 3). However, when comparing the average time across all participants, no significant difference was found (p = 0.342). Second, the analysis of the product selection frequency revealed significant differences between the two conditions. Participants selected lower-priced products significantly more often in the SOC condition than in the OOC condition. On average, participants chose lower-priced products twice as frequently in the SOC condition than in the OOC condition. The paired *t*-test results were statistically significant (t = −19.373,p < 0.001), as shown in Table 4.

## 4. Discussion

This study investigated differences in brain activation patterns and functional connectivity in the PFC induced by two different conditions, OOC and SOC. At the network level, the present findings did not indicate a robust connection of the prefrontal network. Likewise, nodal metrics did not show statistically significant differences after correction for multiple comparisons. By contrast, the seed-based connectivity analysis showed that the connection between the ${ROI}_{4}$ and the ${ROI}_{6}$ was significantly stronger under the OOC condition than the SOC condition. A similar tendency was observed for the connection between the ${ROI}_{4}$ and the ${ROI}_{3}$, although this effect did not survive multiple comparison correction. Together, these findings suggest that the neural difference between the two conditions was not expressed as a broad global network change, but rather as a more limited difference in specific prefrontal connections.

The behavioral findings were partly consistent with this pattern. Participants in the OOC condition tended to spend more time on decisions and selected lower-priced products more often in the SOC condition than in the OOC condition. However, the mean response time did not differ significantly between conditions. Therefore, the results support limited evidence of behavioral tendencies.

### 4.1. Neuroscientific Analysis

The OOC condition requires social cognitive processes that involve the consideration of the uncertain preferences and reactions of others. This may be associated with relatively higher levels of cognitive engagement in the PFC. According to previous studies, complex social cognitive processes induce heightened activation in the PFC, which supports the validity of the increased brain activation observed during the OOC condition in this study [63,64]. Furthermore, other studies have reported that functional connectivity is enhanced during social decision-making processes to integrate emotional elements with cognitive evaluations [65,66].

By contrast, the SOC condition reflects decisions driven by subjective preferences, personal experiences, and a sense of certainty. Consumers tend to make decisions quickly, relying on intuition and the perceived clarity of their choices [67,68]. A previous study by Farrar et al. [69] observed that under certain conditions, there was decreased brain activation in the PFC and simplified functional connectivity compared to uncertain conditions. The present findings indicate that the SOC condition involves a more efficient and straightforward decision-making process, leading to decreased functional connectivity.

The higher brain activation and stronger functional connectivity observed in the OOC condition suggest that cognitive processing may involve considering social relationships with others. The mPFC has been implicated in emotional aspects of social interactions. Because ${ROI}_{4}$ corresponded to a central prefrontal region in the vicinity of the mPFC, the stronger connectivity involving this region under the OOC condition may reflect greater engagement of processes related to considering others’ perspectives. In addition, the differences in brain activation pattern observed in Ch06 are broadly consistent with previous work implicating PFC regions in decision-making and action monitoring, suggesting a link between purchasing and social interactions [70,71]. This finding suggests that consumers may engage in higher-order processes to predict others’ preferences and social expectations when making purchase decisions [72]. These findings suggest that consumer decision-making for others may engage neural processes associated with interpersonal relationships and social cognition.

In addition, this study utilizes fNIRS technology to analyze both brain activation patterns and functional connectivity based on consumer orientation. While previous fNIRS studies have primarily focused on quantitative analyses comparing the activation of brain regions [73,74,75], this study extends the scope of consumer decision-making research by incorporating network analysis using functional connectivity to broaden the understanding of purchasing behavior.

Because fNIRS is noninvasive and has minimal environmental constraints, it has been identified as a suitable tool for conducting multidisciplinary research in real-world scenarios that integrate behavioral economics, social sciences, and neuroscience. This approach can contribute to deepening and expanding the convergence of consumer behavior and neuroscience research.

### 4.2. Limitation and Future Direction

The cortical activation characteristics induced by the OOC condition in this study provided evidence supporting previous findings on social decision-making for others [63,64]. However, this study had several limitations. First, although fNIRS technology indicates excellent performance in identifying brain activation and functional connectivity in the PFC, its ability to analyze the roles of deeper brain regions is limited. To overcome this technical limitation, multimodal neuroimaging techniques, such as fNIRS-fMRI, should be utilized to measure changes in deeper brain areas [76,77]. Second, this study focused on the restricted conditions of consumer intentions. Additionally, the absence of a natural baseline condition makes it difficult to isolate condition-specific neural activity. These limitations make it challenging to analyze the diverse motivations underlying consumers’ purchasing decisions. Future studies should investigate consumer decision-making across various contexts to reflect real-world scenarios. Such research would enable a deeper understanding of the mechanisms underlying consumer decision-making processes.

## 5. Conclusions

This study explored how consumption-oriented attitudes differ in brain activation and functional connectivity using fNIRS technology. These findings provide preliminary neurophysiological evidence that may inform future neuromarketing research and the development of human-centered applications. More broadly, the present findings illustrate the potential of wearable fNIRS as a portable bioelectronic sensing tool for human-centered behavioral applications in realistic decision-making contexts. These results support the utility of wearable fNIRS as a neuromarketing tool for studying consumer decision-making.

## Figures and Tables

**Table 1:** Demographic information summary.

Variable	Category	Number of Participants	Percentage (%)
Gender	Males	25	57
	Females	19	43
Age range (M ± s.d.)	20–30’s	22.84 ± 3.33	100
Employment	Not employed	3	7
	Student	39	89
	Employed	2	4
Education	A high school graduate	30	68
	Bachelor’s degree	14	32
Handedness	Right	41	94
	Left	2	4
	Both	1	2

**Table 2:** Regions of Interest (ROIs) and Seed-Based Connectivity Analysis.

Region	ROI	Channel	Seed-Based Connection	
Lateral right	${ROI}_{1}$	Ch01	S1	${ROI}_{1}-{ROI}_{4}$
		Ch02		
Upper right	${ROI}_{2}$	Ch03	S2	${ROI}_{2}-{ROI}_{4}$
		Ch05		
		Ch06		
Lower right	${ROI}_{3}$	Ch04	S3	${ROI}_{3}-{ROI}_{4}$
		Ch07		
Center	${ROI}_{4}$	Ch08	${ROI}_{4}$ (Seed)	
Upper left	${ROI}_{5}$	Ch09	S4	${ROI}_{5}-{ROI}_{4}$
		Ch11		
		Ch12		
Lower left	${ROI}_{6}$	Ch10	S5	${ROI}_{6}-{ROI}_{4}$
		Ch13		
Lateral left	${ROI}_{7}$	Ch14	S6	${ROI}_{7}-{ROI}_{4}$
		Ch15		

**Table 3:** Statistical comparison between response time under the two conditions. Bold values indicate statistically significant differences * p< 0.05, ** p< 0.01, *** p< 0.001).

Participant	RT Difference (OOC-SOC)	Participant	RT Difference (OOC-SOC)
1	276.47	23	−812.77 **
2	−1706.23 ***	24	768.3 ***
3	752.97 *	25	96.3
4	2555.5 ***	26	−805.6 *
5	1472.5 ***	27	−1336.43 **
6	2112.6 ***	28	−143.5
7	1271.6 ***	29	−107.1
8	−723.87	30	2716.77 ***
9	−672.9	31	803.9 *
10	−2198.2 ***	32	1396.43 ***
11	1992.27 ***	33	−592.83
12	−223.87	34	−1070.4 **
13	4088.1 ***	35	41
14	−1491.7 ***	36	−15.7
15	−740.77	37	−267.37
16	1480.8 ***	38	974.37 *
17	2384.1 ***	39	2206.73 ***
18	1810.03 ***	40	2329.53 ***
19	2935.03 ***	41	−1000.9 ***
20	331.53	42	600.7 **
21	−2924.7 ***	43	−852.47
22	1491.17 ***	44	−694.1 **

**Table 4:** Statistical comparisons of choosing lower-priced products between the two conditions (*** *p* < 0.001; #: number).

Condition	# of Selections	Mean	SD	Ratio (%)	t	df	Significance
OOC	30	7	3.09	23.23	−19.373	29	***
SOC	30	22.97	2.70	76.56			

## Data Availability

All relevant data for this study are publicly available from the GitHub repository (https://github.com/AdvancedICT/Korean_Red_Ginseng (accessed on 13 March 2026)).

## Appendix A

**Table A1:** MNI coordinates and anatomical regions of fNIRS channels with corresponding Brodmann areas (BA) [59].

Channel	MNI			Location	Anatomical Region	BA Label
	x	y	z			
Ch01	54.67	44.00	0.00	Right	Interior Frontal Gyrus	BA46
Ch02	48.00	52.67	12.67	Right	Middle Frontal Gyrus	BA10
Ch03	36.33	58.33	24.67	Right	Middle Frontal Gyrus	BA10
Ch04	39.00	64.67	0.00	Right	Middle Frontal Gyrus	BA10
Ch05	26.33	69.67	12.67	Right	Superior Frontal Gyrus, Dorsolateral	BA10
Ch06	13.67	69.00	24.67	Right	Superior Frontal Gyrus, Medial	BA10
Ch07	14.33	73.00	0.33	Right	Superior Frontal Gyrus, Medial	BA10
Ch08	0.33	68.00	11.33	Center	Superior Frontal Gyrus, Medial	BA10
Ch09	−13.00	68.00	12.00	Left	Superior Frontal Gyrus, Medial	BA10
Ch10	−14.33	73.00	0.00	Left	Superior Frontal Gyrus, Medial	BA10
Ch11	−26.00	68.00	12.00	Left	Superior Frontal Gyrus, Medial	BA10
Ch12	−35.67	57.67	23.67	Left	Superior Frontal Gyrus, Dorsolateral	BA10
Ch13	−38.00	63.00	0.00	Left	Middle Frontal Gyrus	BA10
Ch14	−45.67	51.67	12.33	Left	Middle Frontal Gyrus	BA10
Ch15	−52.33	43.67	−0.33	Left	Orbital Part of IFG	BA47
